# Jaw Functional Orthopedics and Osteopathic Approaches As Adjunctive Treatment for Idiopathic Scoliosis: A Case Report

**DOI:** 10.7759/cureus.94727

**Published:** 2025-10-16

**Authors:** Patricia Valério, Maria Vasilyeva

**Affiliations:** 1 Department of Orthodontics, São Leopoldo Mandic Dental Faculty, Campinas, BRA

**Keywords:** body posture, functional appliance, jaw functional orthopedics, osteopathic, scoliosis

## Abstract

In this case report, we present a patient with idiopathic scoliosis who had been treated with a traditional Chenault corset for seven years without good results. After starting with osteopathy associated with jaw functional orthopedics as adjuvant therapeutics, in 11 months, the difference in posture was evident. It enhanced the effectiveness of the treatment and provided a more complete equilibrium to the patient. Jaw functional orthopedic appliances are able to change the maxillo-mandibular relationship and improve the physiology of the masticatory apparatus, including the position of the condyle inside the articular cavity. Since mandible posture has a great influence on body posture, it can be very helpful as a co-adjuvant therapy.

## Introduction

Over the last 20 years, many articles have been published emphasizing the interface between the stomatognathic system functioning and the body's balance. Musculoskeletal disorders can influence occlusion and temporomandibular biomechanics and vice versa [1-3].

Jaw functional orthopedic appliances can change the maxillo-mandibular relationship and improve the physiology of the masticatory apparatus, including the position of the condyle inside the articular cavity [4]. Osteopathic manipulative treatment has been proven to be effective in the treatment of musculoskeletal disorders [5].

In this case report, we highlight the relevance of using both therapies in an idiopathic scoliosis patient, enhancing the effectiveness of the treatment and providing a more complete equilibrium to the patient. To our knowledge, reports combining osteopathy and jaw functional orthopedics in idiopathic scoliosis are scarce.

## Case presentation

**Figure 1:**
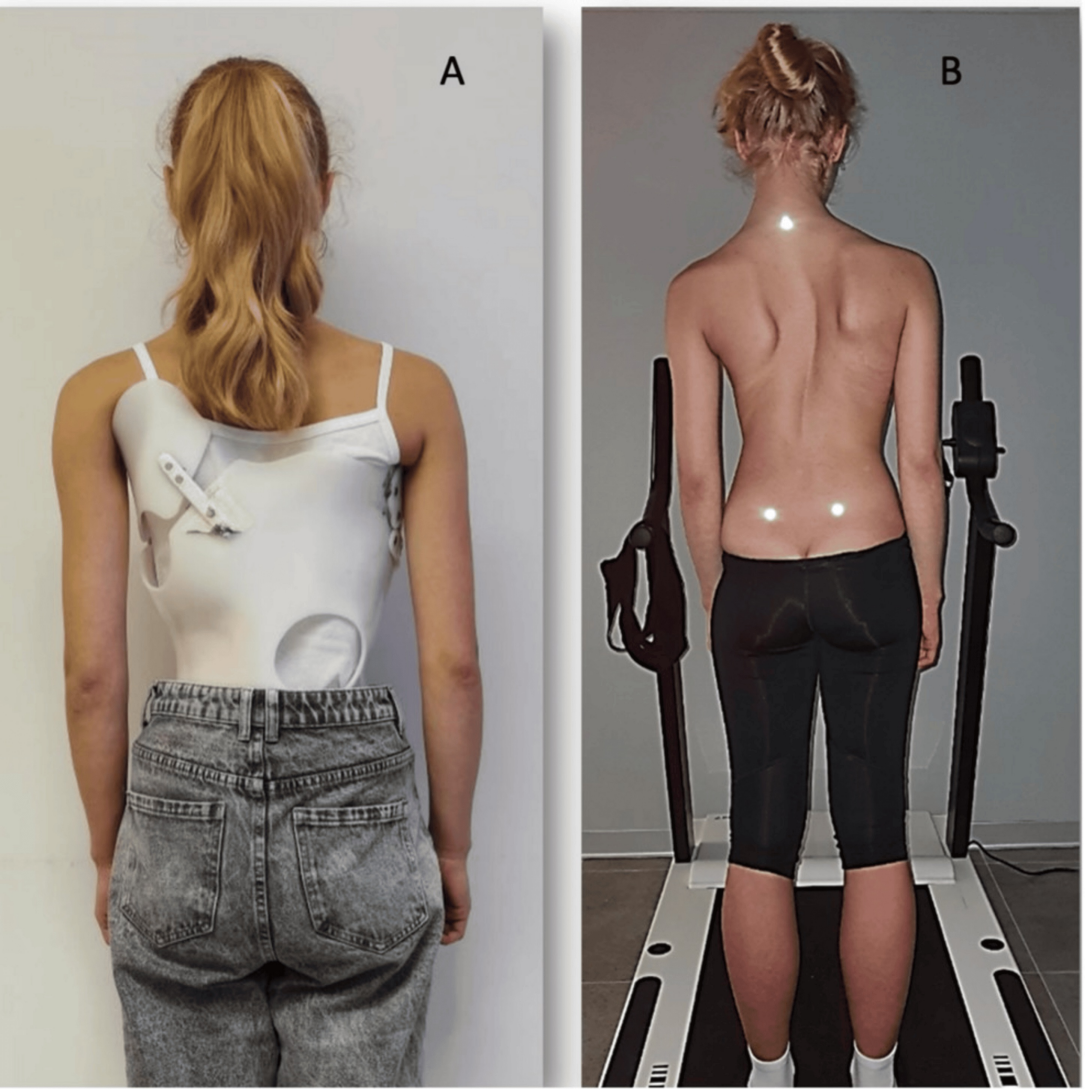
Severe scoliosis. Posterior view of the patient: (A) With corset; (B) without corset.

A 15-year-old girl presented to our clinic with severe scoliosis. The girl was a gymnast up to eight years old when the scoliosis appeared. From that time, she started to use the Chenault corset and stay under the Katharina Schroth method of treatment (Figure 1).

**Figure 2:**
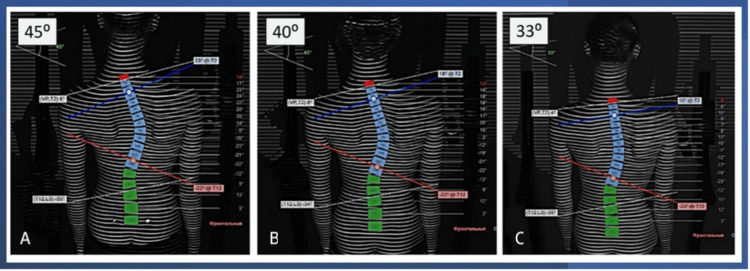
Scoliosis evolution. (A) Before treatment: VP-DM lateral deviation in the thoracic region: 48 mm; vertical deviation: -24 mm; pelvic tilt: 5 mm; kyphosis angle: 22°; lordosis angle: -24°; Cobb angle: 45°. (B) After seven months: VP-DM lateral deviation in the thoracic region: 26 mm; vertical deviation: -19 mm; pelvic tilt: 0 mm; kyphosis angle: 23°; lordosis angle: -22°; Cobb angle: 40°. (C) After 11 months: VP-DM lateral deviation in the thoracic region: 26 mm; vertical deviation: -3 mm; pelvic tilt: 0 mm; kyphosis angle: 16°; lordosis angle: -19°; Cobb angle: 33°. VP-DM: vertebral prominence-dorsal median line distance

However, no significant improvement was observed, and the family tried to search for other specialists. Finally, she attended a consultation with an osteopath (Dr. Grigory S. Perevezentsev), who realized that the stomatognathic system imbalance had an important influence on the maintenance of the scoliosis. He referred the patient to the first author, who is also an osteopath and functional orthodontist (also called a jaw functional orthopedist). The patient started to use a jaw functional appliance created by the author (patent RU221179U1), which provided mandible and tongue posture changes, blocking the lateral movements for some time, aiming to recover the correct functioning of muscles from the spinal and lateral chains, which are directly connected to the stomatognathic system muscles. The improvement in the severity of scoliosis was evident, as shown by the sequence of images (Figure 2).

**Figure 3:**
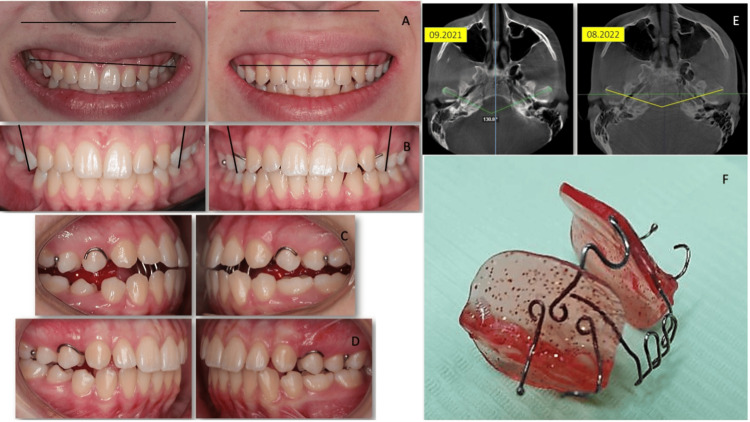
Jaw functional orthopedics treatment. Left panel: Occlusion of the patient before (A, B) and after Jaw Functional Orthopedics treatment (C, D). Right panel: Upper: CBCT images of the temporomandibular joint before and after treatment (E). Lower: Jaw Functional Orthopedics appliance (F). CBCT: cone beam computed tomography

The evolution of occlusion and position of the condyle on the temporomandibular joint, as well as the functional appliance used, is shown in Figure 3.

## Discussion

Static and dynamic body postures are a complex process. For the correct establishment of posture, the integration of afferences from the somesthetic, visual, and vestibular systems to the central nervous system, where information is decodified and generates efferences to adjust posture, is necessary [6]. Idiopathic scoliosis is a deformity without a clear etiology, and in recent years, many researchers have focused on the influence of the stomatognathic system on body posture and specifically the interface between occlusal alterations and scoliosis [7]. A recent study confirmed that there is a connection between malocclusion and scoliosis, suggesting that multi-professional approaches must be adopted, as early as possible, to avoid the progression of the pathology [8]. Another recent study revealed that rapid palatal expansion (a very common procedure adopted in orthodontics where the palatal suture is forced to open using high levels of mechanical force) worsened the degree of scoliosis in patients under orthopedic treatment [9]. Our case reinforces all these points. While therapeutics have focused only on the spine, the results have been poor. As soon as the stomatognathic system was equilibrated, the case completely changed in evolution. Unlike prior studies that have primarily explored rapid palatal expansion or traditional orthodontics, this case illustrates how a non-invasive, functional orthopedic approach, when combined with osteopathy, can lead to measurable postural improvement. Jaw functional orthopedics uses mandible and tongue posture changes to equilibrate the occlusion [10]. The appliance used in this study was constructed according to the protocol described in Appendix A. In addition to traditional methods, DIERS Formetric 4D (DIERS International GmbH, Schlangenbad, Germany) was used to evaluate the improvement in the severity of scoliosis. This examination elucidates, via dynamic images, the changes in posture and has recognized reliability [11]. Mouth and foot evaluations were also extremely important, considering that the first author had previously reported that the inclination of the tibia is closely related to the patient's dental occlusion [12].

## Conclusions

In general, our result emphasizes the necessity of the integration of osteopathic and jaw functional orthopedic approaches in connection with orthopedics to improve the results of the treatment and, consequently, the quality of life of patients with idiopathic scoliosis. This is a single case, and further studies are needed to confirm generalizability.

## Appendices

Appendix A

From Bite to Foot Protocol of Evaluation

This method relates to the field of diagnosis of malocclusion pathology, as well as manual correction of maxillofacial muscles, cranial sutures, postural and foot disorders, before and during orthodontic treatment.

A solution is known from the prior art that describes various diagnostic methods for occlusal-postural abnormalities: RU2709840C2, RU2779362C1.

In the proposed technical solution RU2709840C2, the individual height of the maxillary space is determined by selecting calibrated plates that change the height of the separation of the dentition until the musculofascial tension of the extremities is equalized using the Armstrong manual test sequentially in all three positions: standing, sitting, and lying down.

In the proposed technical solution RU2779362C1, an anamnesis is collected, a dental examination, taking impressions, and a plantography of the feet in the patient's standing position. A 3D cephalometry based on cone beam computed tomography (CBCT), multispiral computed tomography (MSCT) of the head and neck, magnetic resonance imaging (MRI) of the temporomandibular joint (TMJ) in closed and open form, MRI of the spine, orthopantomogram (OPTG), and video recording of gait are performed. Height, weight, and body mass index are determined as anthropometric data. Spirometry is performed, and muscle strength in the arms, heart rate, and blood pressure are determined.

A bioimpedance analysis of body composition is performed, determining the level of water, protein, visceral fat, bone mass, basal metabolic rate, and biological age. Oxygen saturation of the blood, glucose, cholesterol, vitamin D, and vitamins of group B are determined. General physical fitness is assessed - running, jumping rope, squats, push-ups, pull-ups, abs, balance assessment, and a handwriting sample. A Lusher test is performed. After receiving all the information, they are compared with the standard values. The method improves the accuracy of the patient's examination when planning and conducting osteopathic functional dental treatment through a comprehensive assessment of the most significant indicators.
